# Essential Oil Phytocomplex Activity, a Review with a Focus on Multivariate Analysis for a Network Pharmacology-Informed Phytogenomic Approach

**DOI:** 10.3390/molecules25081833

**Published:** 2020-04-16

**Authors:** Alessandro Buriani, Stefano Fortinguerra, Vincenzo Sorrenti, Giada Caudullo, Maria Carrara

**Affiliations:** 1 Maria Paola Belloni Center for Personalized Medicine, Data Medica Group (Synlab Limited), 35100 Padova, Italy; stefano.fortinguerra@gmail.com (S.F.); vincenzo.sorrenti@unipd.it (V.S.); 2 Department of Pharmaceutical and Pharmacological Sciences, University of Padova, 35100 Padova, Italy; maria.carrara@unipd.it; 3 Bendessere™ Study Center, Solgar Italia Multinutrient S.p.A; 35100 Padova, Italy; giada.caudullo@solgar.it

**Keywords:** essential oil, network pharmacology, personalized medicine, phytogenomics, multivariate analysis

## Abstract

Thanks to omic disciplines and a systems biology approach, the study of essential oils and phytocomplexes has been lately rolling on a faster track. While metabolomic fingerprinting can provide an effective strategy to characterize essential oil contents, network pharmacology is revealing itself as an adequate, holistic platform to study the collective effects of herbal products and their multi-component and multi-target mediated mechanisms. Multivariate analysis can be applied to analyze the effects of essential oils, possibly overcoming the reductionist limits of bioactivity-guided fractionation and purification of single components. Thanks to the fast evolution of bioinformatics and database availability, disease-target networks relevant to a growing number of phytocomplexes are being developed. With the same potential actionability of pharmacogenomic data, phytogenomics could be performed based on relevant disease-target networks to inform and personalize phytocomplex therapeutic application.

## 1. From Traditional Use of Essential Oils to Phytocomplex Molecular Characterization

The use of plant extracts dates back to the ancient Mediterranean populations. Aromatic plants, essences, and oils have been used for ages in traditional medicine, ceremonies, beauty care, food preservation, and perfumes. They have also been the basis for herbal and botanical medicines and remedies contributing, together with other traditional medicinal preparations, to the development of pharmaceuticals [1]. The earliest essential oils (EO) usage evidence occurred from 3000 to 2500 B.C. Egyptians are known as the first culture to use aromatic extracts, and essential oils were used in China and India, despite the first evidence of essential oils produced by steam or hydro-distillation seems to be attributed to the Arabs in the Middle Ages [1,2]. Essential oils contain complex mixtures of volatile compounds derived from aromatic plants, mostly composed of terpenes (monoterpenes, sesquiterpenes, etc.) generated by the mevalonate pathways. However, other compounds are present, like phenolics, derived via the shikimate pathway [3,4]. EO components can be synthesized by all plant organs and are stored in secretory cells, cavities, canals, epidermic cells, or glandular trichomes [2,5].

EOs are generally extracted by low/high-pressure distillation. Other processes include solvent extraction, absolute oil extraction, resin tapping, wax embedding, cold pressing, liquid carbon dioxide, or microwaves [6]. Most of the commercialized essential oils are chemotyped by gas chromatography and mass spectrometry analysis. Analytical monographs are available (European pharmacopeia, ISO, WHO, Council of Europe) [7], to ensure consistent EOs quality. Essential oil chemical profile differs importantly depending on climate, soil composition, plant organ, age, vegetative state, and type of extraction, displaying wide variability in the number of molecules and the stereochemical types of molecules extracted. Due to the extreme variability of the essential oils’ chemical profile, the EO biological effects can vary strongly, depending on the quality and quantity of the active molecules in the phytocomplex [4,8]. Standardized conditions of extraction are required for consistency, the same plant organ, growth in the same soil, under the same climate, and collected in the same season and the same time of the day.

EOs are known for multiple biological activities, among which antioxidants, antiseptic, antifungal, analgesic, anti-inflammatory, spasmolytic, and anesthetic properties [4,9,10,11,12] and for their cytotoxic effect on different human cancer cell lines [13,14]. Although numerous molecular mechanisms of action have been proposed for different EOs, most studies have tested purified molecules, making it hard to correlate the biological activity with the mixture of different components of the phytocomplexes. The high variability of the EO chemical composition and the use of not standardized phytocomplexes has often led to different activities, even in the same contexts [15]. Chemical fingerprinting, like metabolomic analysis, is normally used to precisely identify chemical composition and characterize EOs [16].

## 2. Identification and Isolation of Bioactive Compounds and Derivatives from Essential Oils

EOs biological activities are usually tested on pharmacological experimental models, and the activity is normally attributed to the most meaningful molecule(s) based on the composition. Nevertheless, when used alone, the same purified single molecules usually do not possess the same biological activity. This is usually attributed to the presence of many different molecules, many with similar structures, that can collectively affect the biological activity. As a result, pharmaco-toxicological parameters such as IC50, although useful to screen different EOs with respect to specific biological activities [17], can hardly be used for their pharmacological standardization due to the wide variability of EO components. Using the phytocomplexes’ main characteristic molecules or families has been one common strategy to standardize herbal preparations, assuming a linear correlation between the pharmacological activity and the main components of the phytocomplex [18]. The main limit of this approach is the exclusion a priori of significant contributions of the lesser components of the phytocomplex to the biological activity, as well as the biological cooperation between components, thus misrepresenting the multiple component nature of the phytocomplex.

EO research has often used bioactivity-guided fractionation to identify fractions enriched with the pharmacological activity of essential oils or other phytocomplexes, an approach that has often led to the identification of molecules or families further developed to obtain drugs [19,20,21]. Phytocomplexes are progressively fractioned, and the biological activity enriched. Fractions isolation depends on the extraction methods, and in some cases, has led to the purification of some individual components of the essential oil most endowed with the original biological activity. Isolation of single molecules from essential oils follows the logic of the classical reductionist pharmacological approach to identify single compounds associated with a specific activity. This approach has allowed the identification of important bioactive compounds also in EO, as in the case of terpenes. Nevertheless, despite many scientific in vitro and in vivo findings demonstrating the efficacy of single molecules extracted from essential oils such as for thymol, carvacrol, eugenol, beta-caryophyllene, menthol, and pinitol among others, the original bioactivity found in the phytocomplex is often reduced and few clinical studies on humans have demonstrated their clinical applicability [22,23,24,25,26,27,28]. D-limonene is an example of translational failure. Considered a promising antitumoral molecule against many types of cancers, when trialed in the clinic it revealed a lack of efficacy [29,30].

In general, the reductionist, magic-bullet oriented approach of identifying single molecules to strike a pharmacological target, has revealed with time its intrinsic limits when dealing with phytocomplexes. In most cases, the biological activity of the isolated compound does not correspond to the initial biological activity of the phytocomplex, where multiple synergies and antagonisms between molecules and between molecules and molecular targets occur and contribute to the biological activity as a whole [31]. By removing the original natural context, molecule isolation and purification eliminates the very intrinsic nature of the plant-prepared mixture, thus annihilating its multifaced biological activity. Moreover, both experimental models and analytical methodologies designed for pharmacological studies of single molecules, are often not suitable for investigating mixtures of different substances, hampering the full exploitation of the intrinsic potentialities of natural phytocomplexes such as essential oils. Different, more adaptable experimental models and analytical approaches would be more feasible, where multiple molecules and multiple effects can be simultaneously analyzed while maintaining the original context. The use of -omics technologies and a systems biology approach is today a powerful strategy with unprecedented potential for studying phytocomplexes like essential oils in their entirety, taking into consideration all the potentially active components [32,33].

## 3. Oneness and Multiplicity of the Phytocomplex: Pushing Too Far the Reductionist Approach Can Lead to Biological Irrelevance

Herbal products have dominated the pharmacopeia for hundreds of years and have provided large amounts of medicines [31,32,34]. More recently, while the pharmaceutical industry has focused on single drug therapeutics and synthetic drug development, the use of natural products in drug discovery has been reduced. This approach has been favored by the advent of structure activity-guided organic synthesis and large-scale screenings. Synthetic pharmaceutical production reduced the connection between plants and human health, making modern medicine highly dependent on medications mostly based on single molecules endowed with target-specific molecular mechanisms of action [35]. Unfortunately, this reductionist approach, though leading to some of the most important therapeutical breakthroughs, is intrinsically unfeasible for the study of herbal drugs, whose activity is linked to the multiplicity of bioactive components present in the phytocomplex and the corresponding plethora of molecular targets [31]. Complex mixtures of compounds in herbal drugs have been shown to exert stronger effects than the single, isolated compounds [36]. Several trials evaluating whole plant extracts activity versus purified preparations have shown that the potency declines with the progressive fractionation and purification of the mixture [37,38]. The synergistic, cumulative, or the addictive properties, as well as enhanced bioavailability of plant constituents, have been proposed to explain the different effectiveness [39]. Endo-interactions (interactions between substances present within the phytocomplex) and exo-interactions (interactions with other substances encountered in the biological environment of the target organism) may have a profound effect on the pharmacokinetic and pharmacodynamic properties, as well as on the potential toxicological side effects of complex drugs [32]. Indeed any drug, as well as endogenous mediators, even when acting on one single target, can trigger many different biological phenomena depending on the target compartmentalization. Considering the complexity of a biological response to a single mono-active drug, the identification of the herbal drug interactions of each single component with its own molecular target(s) can be particularly challenging [32]. The biological effect of a phytocomplex is the collective effect of all its components, some of which will cooperate and some might modulate, while others will act on different, distantly connected targets, ultimately generating several biological events, most of which will probably never overcome the redundancy threshold of the biological system balance control and not become evident [40].

Diseases with a multifactorial etiology are today increasingly treated using different drug combinations, aiming at different targets (e.g., systemic arterial hypertension, atherosclerosis, type-2 diabetes mellitus, tuberculosis, cancer, infections by multi-resistant bacteria, heart failure, septic shock, etc.) [39]. It is reasonable to assume that a mixture of compounds (phytochemical or synthetic) would have greater bioactivity than a single compound because a mixture of bioactive compounds can affect multiple targets [7,34,35]. Modern medicine has learned how rapidly pathogens and cancer cells can develop resistance to single-ingredient drugs. Administration of complex drug cocktails to circumvent or delay the development of resistance to drugs is today a winning therapeutical strategy. Plants learned this strategy very early in their evolution to survive. By relying on combinations of pleiotropic, multi-targeted molecules, plants may have perfected interacting phytochemical complexes to accomplish many complementary tasks [41,42]. The synergism among single herbal extract compounds are mainly related to two factors: the simultaneous solubility of a group of substances with different polarities, and the multiplicity of targets including enzymes, receptors, ion channels, transport proteins, antibodies, and many others [43]. There is thus a need for the development of new approaches and methodologies for pharmacological studies and clinical trials evaluating the effects produced by complex mixtures of compounds [37,40].

Systems biology integrates information about individual components of a biological system. Large databases from various sources and dedicated software can be used to predict the effects of substances on human health [44]. In network pharmacology, a systems biology approach can reconstruct complex molecular pathways from large datasets, providing the basis for the identification of the links between drugs, biological targets, and human diseases, which would be too challenging to interpret experimentally [44,45]. The creation of complete databases containing information on networks of human protein–protein interactions and protein–disease associations has made this possible. Experimentally determined pharmacological data of a given chemical mixture can be fed into these networks to obtain information on chemical interactions, their links to biological activities, and then to human diseases [44]. This mixed in silico approach is today opening new possibilities to properly study the multicomponent and multitarget effects of phytocomplexes like EO.

## 4. Phytochemical Research, the Emergence of the Holistic Approach

Omics technologies allow the simultaneous detection of entire molecular families in a given biological system. At the same time, bioinformatics provide different software tools to collect, classify, network, and view a large number of analytical data. Systems biology offers the system-level framework and a holistic approach to all biologic phenomena, based on the analysis of molecular networks in their dynamic interactions within highly interconnected pathways [46,47,48,49,50,51,52]. The application of -omics techniques is thus demonstrating to be inherently appropriate for the pharmacological assessment of EO and their multiple biological targets [53,54,55,56].

Phytocomplexes exert their biological activity by influencing the steady-state of a large number of components in a biological system and their interactions. Biomolecules create tightly integrated networks, and biological responses derive from the behavior of such networks [57]. Phytocomplex mediated effects can be envisioned as the net output of changes in the properties of a vast number of molecules, all acting in an interdependent fashion to form a highly connected network. The mutual empowerment of omics and systems biology derived from their combined use, finally allows a view of biological systems as a whole, and thus represents a holistic analytical alternative that is more feasible to study complex mixtures as essential oil phytocomplexes [58,59,60,61].

## 5. Network Pharmacology Meets the Phytocomplex

The multimolecular systems approach of network pharmacology provides a strategy based on bioinformatics tools (databases, software) to map the multiple simultaneous interactions of the meaningful molecular clusters of the phytocomplex with their biological targets, highlighting pharmacologically relevant pathways. By informing the therapeutic potential of the phytocomplex, the analysis of relevant interactions with pharmacological network nodes empowers the actionability of its applications.

This field is currently being extensively used in pharmacology. Identifying in silico the most promising compounds or mixtures of compounds for the desired molecular targets on virtual platforms is currently one emerging strategy in the discovery of new drugs to be used for the treatment of multifactorial diseases [62]. Currently, several research teams have developed in silico platforms at a higher level, endowed with software capable of assembling and analyzing billions of known bioactive compounds that can be used to verify their effectiveness against key proteins associated with multifactorial diseases [63]. Molecular docking programs fit the molecules with the protein in question, to understand if they are able to bind, identifying which, among the many possible molecular orientations of the compound, are most effective. Today it is possible to analyze up to a billion molecules for each of these targets, and some projects foresee the possibility of using increasingly efficient cloud computing platforms [64].

Important results have been obtained with the analysis of herbal preparations from traditional Chinese medicine (TCM), where plants are used as blended herbal medicines in formulas that comprise mixtures of mixtures, with each herbal component supposed to exert its specific role, either as an effector, an enhancer, or a mitigator [32,65]. TCM studies have turned abundantly to network pharmacology to re-interpret this traditional knowledge scientifically. In most cases, components of the phytocomplexes are identified and then correlated to biological activities, based on known molecular associations collected in database libraries. In contrast, the actual biological activity is only verified experimentally as a subsequent step. Although this approach takes into consideration every molecule in the phytocomplex, it relies on informed databases with data from experimental conditions in which the molecules had been used alone or in different mixtures or conditions that may not faithfully represent what happens when herbal medicines are used entirely. A bias is thus generated in the selection of the mechanisms of action of the molecule-target-disease network, which might ultimately mislead the investigator.

These TCM studies are based on knowledge from the traditional use of herbal formulae. In most cases, the evidence is a body of traditional medical observations collected during millennia of practical experience, guided by a holistic philosophical framework. The medical approach focuses on wellness based on maintenance of balance between opposite and complementary principles, linked by a flow of energy (Qi) extending as a continuum from cosmos to individuals. Notwithstanding the non-scientific framework, TCM has been successfully using herbal formulae for centuries, and these are still used in integration with clinical, scientifically sound therapeutic interventions. The recent introduction of omic analytical techniques, endowed with bioinformatics and a holistic systems biology approach, has opened to the possibility of re-interpreting complex TCM herbal formulae within a scientific framework. Indeed in the last ten years, numerous network pharmacology studies have produced a wealth of experimental data that today provides a new interpretation supporting the use of phytocomplexes from TCM formulae [66,67].

**Figure 1:**
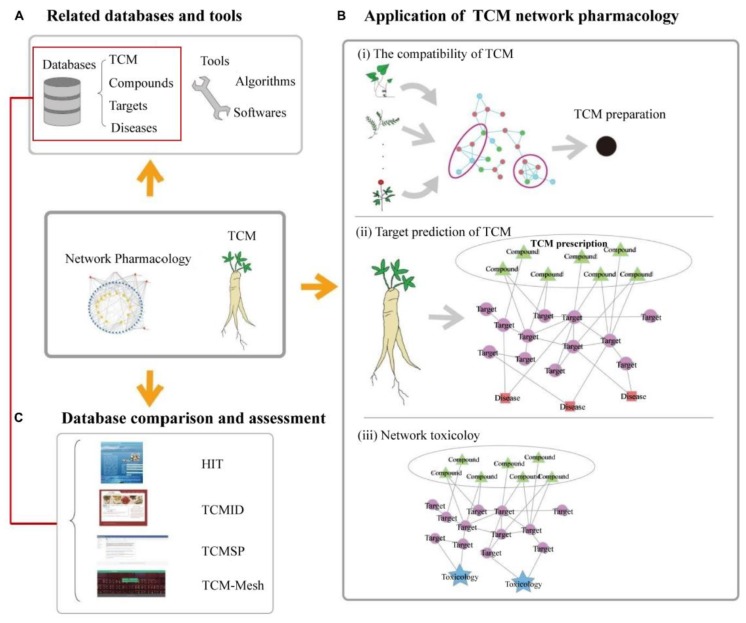
Example of network pharmacology applied to traditional Chinese medicine (TCM) formulae. (**A**) use of databases and bioinformatics for the identification of correlations between TCM, substances, targets and diseases; (**B**) building the pharmacological networks; (**C**) Database comparison and assessment. Distributed under the terms of the Creative Commons Attribution License (CC BY) Copyright © 2019 Zhang, Zhu, Bai and Ning [67].

A network pharmacology approach involves a functional reconstruction of the phytocomplex based on its molecular components. Their association with relevant molecular targets is the first layer of the network, while the further association to disease and pharmacological effects completes the network (see Figure 1 for an example of network pharmacology application to TCM formulae). Even though this approach provides new insights into the molecular mechanisms of phytocomplexes, the sum of the targets affected by the single molecules does not necessarily reflect the global activity of a phytocomplex. It would thus be important to support the use of network pharmacology with experimental data obtained directly from phytocomplexes [68]. This would make it possible to identify targets sensitive to the phytocomplex as a whole.

Evolving from an experience-based medicine to an evidence-based one is still challenging, but network pharmacology has allowed an unprecedented significant scientific growth in the field. Well-structured traditional medicines, with a highly personalized approach and a deep knowledge on preventative strategies, are at the forefront of a new impulse towards integrative methods, aimed at bringing together deterministic and holistic medical traditions [69,70,71,72,73,74,75].

The same approach can be applied to the study of other multitarget mechanisms of action typical of phytocomplexes, including EOs. A critical step to create molecule-target networks involves pharmacodynamic and ADME (absorption distribution metabolism excretion) characterization to verify their bioavailability to reach biologically significant targets. Once drugability has been evaluated, computational methods and databases applied in herbal medicine can be used to identify potential drug targets from multiple therapeutic areas. Identifying drug-target interactions provides essential elements for a network construction where drugs and targets are represented as nodes and the interactions as edges, where all the elements are connected to one or more nodes. The prediction of target profiles and pharmacological actions can then lead to the drug-target-disease co-module associations [32,56,76,77,78,79,80,81,82,83,84]. To date, several studies have been conducted in different clinical areas, from cerebrovascular diseases to neurodegenerative diseases, from cancer to mental illness, internal medicine, and wellness [85,86,87,88,89,90,91,92,93,94,95].

## 6. Analytical Strategies Fit for Studying Phytocomplexes

Essential oils can differently synergize, antagonize, and/or interact with the human body by numerous mechanisms. Their pharmacological efficacy is obtained thanks to integrated multimolecular systems that analyze different biological phenomena leading to a collective effect of clinical significance [96]. Herbal medicinal products or herbal preparations, including essential oils, play an increasingly significant role in healthcare, as preventative medicines, nutraceutical, health foods, and natural health products [97]. The composition of herbal products is highly variable, and their standardization cannot be easily achieved, as would be required for clinical use. Moreover, understanding their complex molecular mechanism of action is pivotal for a proper pharmacological use, an aspect that encounters many technical problems at different stages, from pharmaceutical standardization to consistency of effectiveness. Various genetic and phenotypic characteristics, growth conditions, and the manufacturing chain can account for variations seen in the plant metabolome. Given that the nature and function of bioactive constituents in herbal preparations are usually not well understood, a satisfactory quality control of herbal preparations is often missing [98]. Standardization of herbal drugs based on constituents with known therapeutic activity is commonly used, but this can lead to a bias in the quality evaluation with the reductionistic assumption that single components are solely responsible for the therapeutic efficacy. The identification and assessment of the contribution of all components and their interactions to the pharmacological effect requires the application of advanced analytical and high-content technologies, including “omics” methods, computational modeling and simulation approaches, and, most of all, a holistic vision, and specifically, a systems biological thinking [85,99].

Methods of multivariate statistics can evaluate chemical fingerprints to classify samples and predict their quality. Chemometric methods can be used for assessing data relating to the quality of herbal products [100]. Techniques include principal component analysis (PCA), local least square (LLS), linear discriminate analysis (LDA), spectral correlative chromatography (SCC), heuristic-evolving latent projections (HELP), information theory (IT), and orthogonal projection analysis (OPA). Other methods can be used to provide key information for building networks and connections, like Bayesian networks and graphical models (e.g., Markov random fields) [101]. Assessing the quality of herbal drugs from a combined metabolomic-bioactivity profile perspective seems to be the most appropriate approach to capture the relationships between multiple constituents and synergisms, to help understand the active components and their mechanisms of action [99]. These methods play a key role in the quest for active ingredients of essential oils, allowing their analysis as clusters within the context of the phytocomplex, therefore allowing the building of molecule-target networks that reflect the real type and level of biological activity experimentally observed.

## 7. Multivariate Approach to Study Essential Oils’ Biological Activity

**Figure 2:**
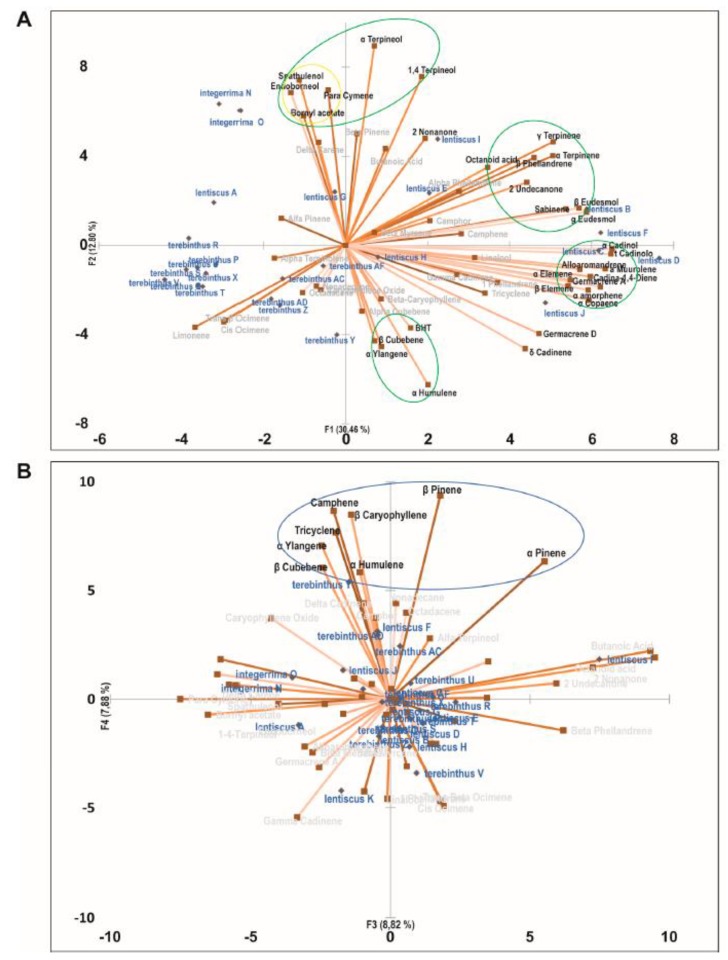
Principal Component Analysis of the cytotoxic effect of Pistacia essential oils on LoVo cells. (**A**) principal component analysis (PCA) biplot with PC1 and PC2 distribution of essential oil samples and chemical components of the phytocomplexes. (**B**) PCA biplot with PC3 and PC4 distribution of essential oil samples and chemical components of the phytocomplexes. Clusters of cooperating compounds with a positive correlation to one or two components are identified with circles (green for *P. lentiscus*, yellow for *P. integerrima*, and blue for *P. terebinthus*). Taken with permission from Buriani et al. [68].

There are only a few examples where essential oil biological activity has been associated with its components directly from crude experimental data, a purely inductive methodology, as like most classical experimental models. Our group used gas chromatography analysis coupled with mass spectrometry (GC/MS) and principal component multivariate analysis (PCA) to study the cytotoxic activity of essential oils from various species of the *Pistacia* genus on human tumor cell lines. The biological activity of different samples from various species of *Pistacia* was plotted versus the molecular fingerprint of the EOs, and several clusters of molecules resulted in associating significantly better with the biological activity. In particular, PCA was performed on a Pearson correlation matrix, computed with the contribution of each compound to the IC50 of each oil sample, taking into consideration the intrinsic contribution of the compound to the cytotoxic effect of the phytocomplex. PCA allowed the identification of 46 compounds in the phytocomplexes correlated with potential biological activity, distributed within different clusters of molecules potentially cooperating to achieve the cytotoxic activity on the cell lines. The analysis does not take into account the contribution of the single molecules, but the final result of their presence in the biological environment, providing an inductive, and at the same time, holistic reading of the experimental evidence. Merging the chemical composition data and the biological results by a multivariate approach allows evaluating the bioactivity of complex mixtures. At the same time, it highlights the cooperating clusters of bioactive molecules (see Figure 2) [68]. The graphical display of the correlation matrix, obtained through PCA, also allows to display patterns and interpretative schemes capable of making hypotheses on the possible activities of the cluster of substances present within the phytocomplex.

## 8. Network Pharmacology Guided Phytogenomics for Personalized Medicine

Once significant clusters of bioactive molecules are identified within the framework of their original poly-molecular complex, the appropriate analytical steps need to be pursued to translate the molecular knowledge into a potential therapeutic context. The best possible characterization is needed for direct and indirect molecular mechanisms of action that together can contribute to a therapeutic effect. As previously suggested, this step can be achieved using a network pharmacology approach, with the construction of the phytocomplex-target-disease network based on the known molecular targets of the experimentally bioactive molecules in the EO. With the characterization of the disease-relevant target network, it is possible to rationalize the multiple effects of the essential oil components maintaining the integrity of the poli-molecular nature of the phytocomplex and standardize its use to treat or prevent specific medical conditions. The therapeutic actionability of this information finally depends on the experimental strength of available data on the phytocomplex, so that proper clinical trials can be carried out.

**Figure 3:**
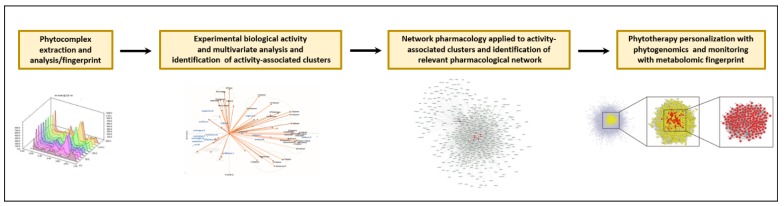
Proposed workflow for molecular characterization, pharmacological activity, and therapeutic application of essential oils phytocomplex: from single molecules analysis to multivariate approaches and network pharmacology to phytogenomic personalization.

Individual genetic profiling is becoming more common in the clinic and pharmacogenomic data can be used to personalize therapeutic interventions. Pharmacogenomics is used to identify key molecular assets of pharmacological interest, providing essential pharmacokinetic, pharmacodynamic, and toxicological information. Personalization of therapies can be based on the genetic characteristics of the individual, affecting how drugs are absorbed, distributed, metabolized and excreted, how the pharmacological targets respond to treatment, and how susceptible the individual is to toxic effects. Individual molecular characteristics can thus be identified for prominent risk factors and treatments tailored using targeted therapies. The identification of networks between phytocomplexes and targets highlights the molecular mechanisms that collectively are associated with the herbal effect and depend on molecules whose genetics can significantly affect the efficacy of the herbal treatment [86]. Different individuals with different genetic variations in such molecules will likely respond differently to the phytocomplex. Knowing the individual genomic assets concerning the molecular networks implicated in a given herbal effect will allow a better choice and a more proper regimen for therapeutic or preventive treatments, providing a phytogenomic individual profile. Genetic profiling and a pharmaco-toxicological characterization of the patient could be performed before prescribing or administering herbal products, thus allowing a personalization of its use [79,102,103]. Thanks to their high sensitivity and analytical potency, metabolomic procedures, analyzing the dynamic changes of endogenous metabolites in vivo after administration of herbal medicines, have been utilized to examine biological fluids and monitor phytocomplex administration. Although the high variability of the analytes can still be an obstacle to the standardization of the phytocomplex-biofluids metabolome, this methodology can provide phytocomplex-specific biomarkers that can be used to monitor treatments, and represent an effective diagnostic-omic approach for the evaluation of the effectiveness of a personalized phytotherapeutic intervention [104,105,106]. Collectively, the advances of systems medicine and network pharmacology, together with phytocomplex-related pharmacogenomics, provide new potential strategies and tools for a guided and assisted use of phytocomplexes (see Figure 3 for a schematic representation of the potential applicability line for EO phytocomplexes).

In conclusion, the high-throughput power of omic disciplines together with bioinformatics, multivariate analysis, and in silico methodologies, rationalized within the holistic framework of network pharmacology, provide a fast-growing and unprecedented number of tools and a new strategy to study multifactorial biological environments. This introduces a multidisciplinary scientific approach to study complex mixtures that have long been approached with an experience-based view or a reductionist single active-molecule isolation quest. Currently, it is becoming increasingly possible to bridge together direct experimental data with the real multimolecular composition of biologically active mixtures, thus contributing to an evidence-based study of phytocomplexes. Such an improved insight into phytocomplexes can promote the development of drugs based on essential oil bioactivities, as well as new strategies for activity-driven drug development based on multi-target and molecular cooperation of drug combinations. In this context, the pharmacological activity of known drugs can be improved by the addition of other molecules, thus guiding the conception of new multimolecular drugs, exploiting their synergy in multi-drug combination therapies, or multitarget drugs [65,107,108,109,110,111,112].
